# Direct-to-Implant Extracellular Matrix Hammock-based Breast Reconstruction; Prepectoral or Subpectoral?

**DOI:** 10.1186/s13063-020-4125-6

**Published:** 2020-02-10

**Authors:** Diana L. Dyrberg, Gudjon L. Gunnarsson, Camilla Bille, Jens A. Sørensen, Jørn B. Thomsen

**Affiliations:** 1 Department of Plastic Surgery, Odense University Hospital, Odense/ Lillebaelt Hospital, Vejle, Sdr Boulevard 29, 5000 Odense, Denmark; 2 Department of Plastic Surgery, Telemark Hospital, Skien, Norway; 3 Department of Plastic Surgery, Odense University Hospital, Odense, Denmark

**Keywords:** Breast animation deformity, Immediate breast reconstruction, Breast implants, Subpectoral implant placement, Prepectoral implant placement

## Abstract

**Background:**

Skin-sparing mastectomy followed by immediate implant-based breast reconstruction is a commonly used treatment for breast cancer. However, when placing the implant in a subpectoral pocket, a high incidence of breast animation deformity (BAD) has been reported. Besides the nuisance that BAD can cause, lifting of the pectoralis major muscle (PMM) can result in a more extended postoperative recovery period. When placing the implant solely prepectorally leaving the PMM undisturbed, the incidence and severity of BAD might be mitigated. However, new challenges may occur because of thinner skin cover.

**Methods/design:**

A prospective, multi-centre, randomised controlled trial will be carried out with the primary aim of assessing and comparing the incidence and degree of BAD in women having a direct-to-implant breast reconstruction with either a prepectorally or a subpectorally placed implant. The secondary outcomes are shoulder and arm function, quality of life, aesthetic evaluation, length of stay, complications, need for surgical corrections, and development of capsular contracture. A total of 70 included patients will be followed under admittance and at clinical check-ups 3 months and 1 year after surgery.

**Discussion:**

To our knowledge, this trial is the first randomised controlled trial evaluating and comparing subpectoral and prepectoral implant placement when performing direct-to-implant breast reconstruction following skin-sparing mastectomy. The results will hopefully provide us with a broader knowledge of the outcomes of immediate breast reconstruction, making better preoperative planning possible in the future by providing our patients with a more objective information.

**Trial registration:**

ClinicalTrials.gov, ID: NCT03143335. Prospectively registered on 8 May 2017.

## Background

Mastectomy is frequently used in breast cancer treatment and increasingly performed prophylactically as a risk-reducing intervention as either a skin-sparing mastectomy or a nipple-sparing mastectomy. Due to recent technical advances, the mastectomy procedure is more conservative than before and increasingly allows for immediate breast reconstruction [1, 2]. In immediate breast reconstruction, an implant is often placed below the pectoralis major muscle (PMM) caudally supported by a biological or synthetic mesh [3–5]. The hammock method was introduced by Salzberg and Breuing more than a decade ago [6–9].

Breast animation deformity (BAD), also referred to as breast distortion or ‘jumping breast’, is characterised by an unsightly deformation of the whole breast, breast skin or nipple-areolar complex [10]. The consequences of BAD have been described in cosmetic surgery following subpectoral breast augmentation by Spears in 2009 [11] although have not been widely addressed until recently.

Spears evaluated 40 breast-augmented women with a subpectoral positioning of the implant, which revealed that 77.5% had some kind of distortion during PMM contraction [11]. The theory is that BAD occurs due to the pressure applied by the contracting PMM on the underlying implant. There is a reason to believe that the severity of BAD may be more pronounced in women having a direct-to-implant breast reconstruction; there is less soft tissue to camouflage the muscle and underlying implant.

Breast reconstructions using subpectorally placed implants have been the mainstay until 2014 when prepectoral implant placement in combination with a synthetic titanium-coated mesh was introduced [12]. In prepectoral breast reconstruction, the implant lies above the PMM without disrupting the muscle. The aesthetic outcome of prepectoral implant-based breast reconstruction is comparable to the subpectoral techniques [13, 14]. The question is whether the PMM is needed for implant-based breast reconstruction. Data comparing the cosmetic and functional outcomes of prepectoral or subpectoral breast reconstruction is scarce. It seems that BAD may be prevented by placing the implant in the prepectoral plane. However, this has not been tested in a randomised clinical trial.

In this study, we compare the degree of BAD in patients having a direct-to-implant breast reconstruction using either subpectoral or prepectoral implant placement. The primary outcome measure of our study is the degree of BAD, assessed by the NSE scale [15]. There are currently other trials ongoing concerning immediate breast reconstruction and prepectoral placement of the implant. The trials are investigating different primary outcomes to our study. The trial with ClinicalTrials.gov identifier NCT02830685 aims to test whether there is a difference in outcomes between biological and synthetic mesh when placed as a support under the skin flaps when performing direct-to-implant prepectoral breast reconstruction. The primary outcome measures are surgical complications and technique failure. The trial with identifier NCT02831426 aims to test whether there is a difference in the outcomes between using biological and synthetic mesh when performing two-stage tissue-expander prepectoral breast reconstruction. The primary outcome measures are surgical complications and technique failure.

Furthermore, a breast reconstruction evaluation study is running; ISRCTN11898000, evaluating the safety of prepectoral breast reconstruction with the primary outcome measure being implant loss rate at 3 months.

The secondary aims in our study are to assess the functional and cosmetic outcomes between subpectorally and prepectorally reconstructed groups and to evaluate the patients-related outcome.

## Methods/design

### Study objectives

The primary outcome of this randomised controlled trial is the degree of BAD, assessed by the NSE scale [15]. The degree of BAD, will be assessed by two plastic surgeons viewing videos recorded at 12-month follow-up. In cases of disagreement, a consensus will be reached.

The primary objective of this study is to compare the degree of BAD between two groups of patients having a direct-to-implant breast reconstruction, randomised to either subpectoral implant placement or prepectoralimplant placement. The secondary objectives and comparisons between groups are: (1) assessment and comparison of the shoulder and arm function by use of the Constant Shoulder Score (CSS), (2) assessment and comparison of quality of life (QOL) by Breast-Q, (3) assessment of postoperative pain by the patients during the first three postoperative days using a visual analogue scale from 0 to 10, (4) comparison of time to discharge, (5) comparison of time for surgery, (6) registration and comparison of complications, major and minor, (7) aesthetic outcome evaluated by two consultant plastic surgeons, (8) assessment and comparison of the degree of capsular contracture and (9) identification of new breast cancer and breast cancer recurrence after 3 years registered in the National Patient Registry [16].

### Design of the study

**Fig. 1:**
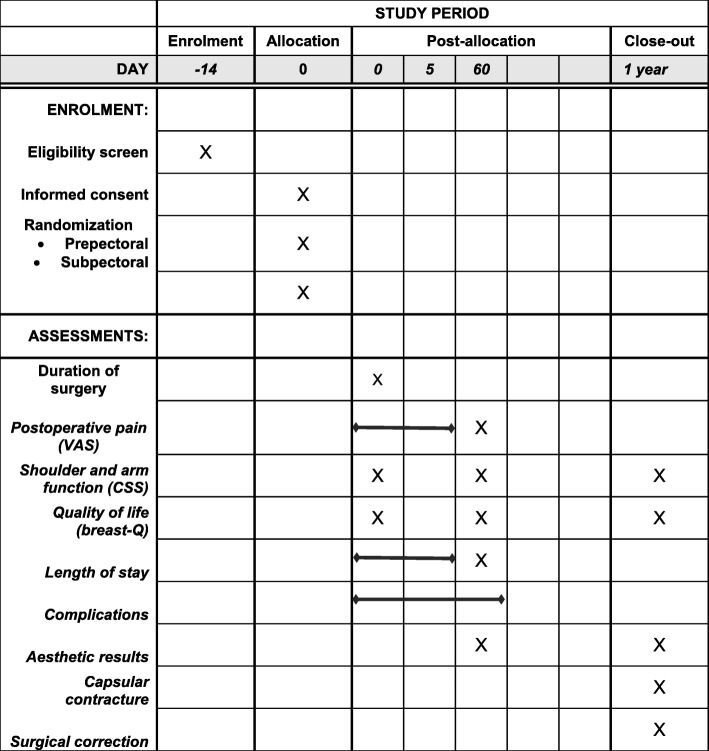
Participant timelines

This study is a prospective, randomised, multi-centre trial with two arms. After giving informed consent to participate in this study, patients are randomised to either subpectoral or prepectoral placement of the implant. In bilateral cases, both breasts are randomised as one case. All included patients will be followed under admittance and at clinical check-ups after 3 months and 1 year of reconstruction. Trial participants will not receive any compensation or remuneration for their participation in the trial. We will conduct the protocol of this trial according to the Standard Protocol Items: Recommendations for Interventional Trials (SPIRIT) guidelines (Additional file 1) [17]. The trial schedule is shown in Fig. 1

### Sample size

A non-parametric calculation of the sample size was made based on our assumptions and experience regarding the degree of BAD in the two groups. We used Fisher’s exact test for small sample sizes. Based on our experience we expected that approximately 60% of the patients who are reconstructed using subpectoral implant placement would suffer from BAD as opposed to 20% of the women who are reconstructed using prepectoral implant placement. By using these assumptions in combination with a significance level of 0.05 (one-sided) and a power of 0.95, the total sample size was calculated to 70 patients, 35 women in each group.

However, a retrospective cohort study in women over 18 years of age who had a unilateral or bilateral direct-to-implant breast reconstruction between November 2011 and December 2017 suggested the above sample size assumptions to be too conservative [15]. Therefore, we will conduct an interim analysis with 60% of the patients included (i.e. 0.6 × 70 = 42). The primary hypothesis will be tested conservatively with *n* = 42 patients applying an O’Brien-Fleming type α-spending function (i.e. α_1_^*^ in [18]), resulting in a significance level of 0.0114 at interim and securing an experiment-wise type 1 error of maximal 0.05.

The sample size is calculated using STATA, version 14.0 (StataCorp, College Station, TX, USA) using a two-sample proportion test.

### Research ethics approval and data management

This trial has been approved by The Regional Committees on Health Research Ethics (S-20160160) and registered with the Danish Data Protection Agency (17/13640).

Required information on each participant is recorded electronically in a secure REDCap database [19]. The entered data will be stored on a secure server in The Region of Southern Denmark via Odense Patient Data Explorative Network (https://open.rsyd.dk).

### Study setting

This study is a collaboration between three surgical centres: (1) the Department of Plastic Surgery, Odense University Hospital, Denmark, (2) the Department of Plastic Surgery, Lillebaelt Hospital, Vejle, Denmark and (3) the Department of Plastic Surgery, Telemark Hospital, Skien, Norway.

### Eligibility criteria

We will invite women aged over 18 years to participate in the study if they are eligible for direct-to-implant breast reconstruction. Patients are recruited by either a plastic or breast surgeon in the outpatient clinic. Patients are excluded based on the following criteria:

- Prior or planned radiation therapy to the breast
- Tobacco usage
- Hypertension treated with more than one drug
- Breast ptosis > 2 measured by Regnault’s ptosis scale [20]
- Body Mass Index (BMI) < 22 or > 32
- Patients having dementia or any psychiatric disorder, making them incapable of providing informed consent or adherence to follow-up and patients unable to communicate in Danish or English

Patients will receive written information, and if needed, each patient is offered a follow-up conversation together with an assessor. Each patient will have time for reflection prior to deciding if she wants to accept or decline to participate in the study. Patients can only be included after written and oral consent to participate in the study.

### Interventions

We plan to include women scheduled for skin-sparing mastectomy as well as nipple-sparing mastectomy. We perform the skin-sparing mastectomy through a periareolar incision. The nipple-sparing mastectomy is performed through an inframammary-crease incision.

For subcutaneous dissection, we use hydro-dissection (1 l NaCl/1 mL epinephrine). When dissecting the gland of the PMM, we use monopolar cautery as we have described previously [7].

### Mastectomy and randomisation

The mastectomy flaps are assessed by the surgeon during surgery for thickness and viability before randomisation. If the flaps are viable and of sufficient thickness, the patient is included and randomised between the direct-to-implant prepectoral and subpectoral implant placement groups. In this study, we use a simple randomisation process. Patients found eligible for direct-to-implant breast reconstruction and who meet the criteria of inclusion will be randomly allocated to either subpectoral or prepectoral implant placement. The randomisation sequences will be generated in advance and stored in sealed envelopes. We will store the sealed envelopes in a secured locker, and the random number table will be kept confidential by the full-time project-responsible person. After randomisation, the allocation will be revealed to the patient only. Blinding post surgery is not possible.

### Subpectoral reconstruction

We divide the PMM insertion infero-medially using monopolar cautery. The division will allow for partial muscle coverage of the implant. The inferior part of the implant is covered by an acellular mesodermal matrix (AMM). This matrix is sutured to the edge of the muscle and the inframammary crease.

We have described the reconstructive technique described in an earlier publication [7].

### Prepectoral reconstruction

The technique used has been published in a paper about prepectoral direct-to-implant breast reconstruction in which we use a single sheet of AMM to cover the implant. The technique is similar in this study, apart from the fact that we have chosen to use two sheets of AMM for full implant coverage [21].

## Follow-up and data collection methods

Pre-surgical data will be collected in the outpatient clinic the day before surgery. Clinical follow-up data will be collected prospectively at 3 and 12 months. We will register all data in a Case Report Form. For registrations of events outside the scheduled follow-up, patients will contact the project responsible person by telephone.

### Outcomes

Before surgery and randomisation, baseline data will be collected in the outpatient clinic. We will record information on age, co-morbidity, medications taken, smoking status, alcohol consumption, whether diabetic, BMI and adjuvant chemotherapy.

The primary outcome measure is the degree of BAD assessed by the NSE scale [15]. Two plastic surgeons will assess the degree of BAD by viewing videos recorded at 12-month follow-up. In cases of disagreement, a consensus is reached. Videos will show each participant in a standing position, relaxed and then performing maximal contraction of the PMM by pressuring the palms of their hands together in the midline in front of their waist. In bilateral cases the most severe side will be used for comparison.

Timeframe: 3 and 12 months.

### Secondary outcome measures

#### Shoulder and arm function

Measurement of the muscular strength of the PMM and deltoids will be examined both before and after surgery. For this purpose, we have chosen the functional assessment tool the ‘Constant Shoulder Score (CSS)’ [22]. This test allows us to make a quantified evaluation of the following four parameters: level of pain, level of function in everyday life, range of movement and strength. We will evaluate the shoulder function bilaterally.

Timeframe: before surgery, and 3 and 12 months after surgery.

#### Quality of life

We will assess the QOL before and after surgery by the reconstructive module of Breast-Q [23].

Timeframe: before surgery, at 3 and 12 months after surgery.

#### Postoperative pain

We will assess the level of pain experienced by the patient at the day of surgery and the three following days by a visual analogue score (VAS) from 0 to 10 where 0 is no pain at all and 10 is worst pain possible.

Timeframe: post surgery and at 3 months after surgery.

#### Length of stay (LOS)

Inpatient days for each patient are recorded as well as days until removal of drains.

Timeframe: number of days until discharge and removal of drains.

#### Duration of surgery

The time of surgery for the two reconstructive methods will be recorded and compared.

#### Complications

We will record any incidence of complications: (1) skin necrosis, (2) wound dehiscence, (3) infection, (4) seroma formation, (5) bleeding and (6) explantation of the implant. Complications will be classified as either major or minor depending on the need for surgical revision in general anaesthesia.

Timeframe: 3 and 12 months after surgery.

#### Aesthetic results

The cosmetic outcome is evaluated by two independent plastic surgeons as well as by the patient [24, 25]. For this purpose, we use a visual analogue scale (VAS) from 0 to 10 were 10 is the best possible outcome.

Timeframe: 3 and 12 months after surgery.

#### Assessment of photographs

Photographs will be evaluated by two independent consultant plastic surgeons and compared to the cosmetic assessment by the patient.

Timeframe: when follow-up is completed.

#### Capsular contracture

The incidence of capsular contracture will be evaluated and classified according to Baker [26].

Timeframe: 12 months after surgery.

#### Surgical corrections

We will record the number of post-reconstructive fat-grafting and surgical corrections within the first year after reconstruction.

Timeframe: 12 months after surgery.

#### New breast cancer and breast cancer recurrence

We will identify the number of new breast cancers developing in the patients who have undergone a prophylactic mastectomy, and the number of breast cancer recurrences in the therapeutically operated patients. We will investigate this 3 years post surgery reviewing the National Patient Register [16].

Timeframe: 3 years after surgery.

### Statistical analysis

We will conduct all our analysis using STATA (StataCorp, College Station, TX, USA). We use baseline variables to describe characteristics of the trial participants. Continuous variables are expressed as mean and standard deviations or as median and interquartile range (25th to 75th percentiles) if the distribution is asymmetrical. Categorical variables are summarised as numbers and percentages. We will compare the categorical variables between groups with a chi-squared test or Fisher’s exact test depending on the number of events. We will compare the continuous variables between groups using an unpaired *t* test or a Mann-Whitney *U* test depending on data representation. A two-sided *p* value of less than 0.05 will be considered significant and reported with a 95% confidence interval. The consistency between different surgeons’ evaluations of BAD and aesthetics will be evaluated using kappa statistics testing the inter- and intrarater reliability [27]. The primary outcome is the proportion of patients with BAD in the subpectorally and prepectorally reconstructed groups. We will compare between groups using the chi-squared test or Fisher’s exact test and a *p* value of less than 0.05 will be considered significant.

## Discussion

The purpose of this trial is to compare the degree of BAD in two types of direct-to-implant breast reconstruction, using either subpectoral or prepectoral implant placement. No study has documented one method to be superior to the other; also, that one should have fewer side-effects or higher complications rates. We expect the two methods for direct-to-implant breast reconstruction to result in comparable cosmetic results, and we also expect the complication rates and the number of re-operations to be the same in the two groups.

We do acknowledge that the target effect size of this study is large, which entails that the sample size is rather small. The small sample size is a limitation. The sample size is based on our experience using the two different reconstructive techniques and the results of a recent retrospective study. In this study, a significant difference between the degrees of BAD in patients having a direct-to-implant breast reconstruction using either subpectoral or prepectoral implant placement was shown [15].

The apparent advantage of a subpectorally placed implant is the larger volume of soft tissue for coverage. However, the PMM seems to be the main contributing factor for the development of BAD and, maybe as a consequence, harm the shoulder and arm function. The use of the PMM may also influence the degree of postoperative pain as perceived by the patient. It has been described that prepectoral implant placement can eliminate or reduce the degree of BAD and is associated with a quicker postoperative recovery. The disadvantages of the prepectorally placed implant may be an inferior cosmetic outcome due to thin tissue coverage and implant visibility as well as a higher risk of capsular contracture [4, 9]. A meta-analysis from 2016 evaluating more than 17,000 implants found that the risk of significant capsular formation increased more than two-fold with subglandular placement of the implant compared to subpectoral in augmented women [28]. However, the risk of capsular contracture may be less when an acellular dermal matrix (ADM) or an AMM is used [4, 29]. We believe that we can overcome the possible risk of implant visibility and poor aesthetic outcome by careful patient selection, and it has not been our clinical observation so far that the prepectoral implant placement is inferior. However, the thickness of the skin flaps is mandatory for selection of patients for prepectoral implant placement, which is why we have chosen to exclude women with a BMI of less than 22 [30].

The most important assessment is the patient’s QOL. Several papers describe that QOL improved in women after breast reconstruction, including immediate breast reconstruction using an implant and an ADM [25, 31–33]. By evaluating patient satisfaction with the two reconstructive procedures, we can see if there is a difference between groups. A surgeon might perceive BAD, implant visibility and cosmetic results as a problem, but this does not mean that it affects the patient’s QOL.

By investigating all these parameters, we expect to obtain a more comprehensive knowledge of the outcomes of direct-to-implant breast reconstruction, performed by one of these two surgical techniques. It can help us to determine whether prepectoral implant placement may represent a better and gentler method for reconstruction of the breast with lower morbidity than subpectoral implant placement. In all cases, the results of this trial should enable us to provide our patients with better and more objective information before they are subjected to immediate breast reconstruction.

### Trial status

The trial is currently enrolling patients. Recruitment began on 1 April 2017 and is expected to be completed by 1 April 2020.

## Supplementary information

**Additional file 1.** Standard Protocol Items: Recommendations for Interventional Trials (SPIRIT) 2013 Checklist: recommended items to address in a clinical trial protocol and related documents*.

## Data Availability

The datasets generated during the current study are available from the corresponding author and trial responsible author on reasonable request.

## Abbreviations

AMM: Acellular mesodermal matrix
BAD: Breast animation deformity
PMM: Pectoralis major muscle
QOL: Quality of life
